# Complete Genome Sequence of the Probiotic *Bifidobacterium thermophilum* Strain RBL67

**DOI:** 10.1128/genomeA.00191-13

**Published:** 2013-05-02

**Authors:** Christoph Jans, Christophe Lacroix, Rainer Follador, Marc J. A. Stevens

**Affiliations:** Laboratory of Food Biotechnology, Institute of Food, Nutrition and Health, ETH Zurich, Zurich, Switzerland

## Abstract

*Bifidobacterium thermophilum* RBL67, an isolate from infant feces, exhibits bacteriocin-like antimicrobial activity against *Listeria* spp. and *Salmonella* spp. and protects HT29-MTX cells against *Salmonella* infection. Here, the complete genome sequence of the probiotic *B. thermophilum* strain RBL67 is presented.

## GENOME ANNOUNCEMENT

*Bifidobacterium thermophilum* belongs to the *Bifidobacterium boum* group of bifidobacteria (1), which has not been studied extensively. Similar to the *Bifidobacterium longum* and *Bifidobacterium adolescentis* species, *B. thermophilum* strains have been isolated from bovine rumen, calf feces, sewage, and piglet feces (2). In contrast, *B. thermophilum* strain RBL67 was isolated from baby feces in a consortium with *Pediococcus acidilactici* UVA1 (3, 4). RBL67 is a moderately oxygen-tolerant strain that reaches high cell numbers in fermentation (2). Furthermore, it produces a bacteriocin-like inhibitory substance (BLIS) that is active against *Listeria* spp. (4, 5). Clear activity against *Salmonella enterica* subsp. *enterica* serovar Typhimurium establishment and infection was observed in a combined colonic fermentation using immobilized child fecal microbiota and an epithelial HT29-MTX cell model (6, 7). RBL67 increased the life span of the small soil nematode *Caenorhabditis elegans* during *S. enterica* subsp. *enterica* serovar Virchow N90 exposition (6) and reduced the severity of rotavirus-associated diarrhea in suckling mice (8). Its protective and antimicrobial effects, growth characteristics, and technological ability make RBL67 a promising microbe for enhancing gastrointestinal health.

The genome of RBL67 was sequenced using a combined Roche GS-FLX Titanium and Illumina HiSeq 2000 approach (GATC-Biotech, Konstanz, Germany). DNA was prepared with a lysozyme/mutanolysine-based cell lysis and subsequent purification using the Wizard genomic DNA purification kit (Promega, Madison, WI) (9).

RBL67 is the first strain of the species *B. thermophilum* to be completely sequenced, assembled, and publically available. The genomes of closely related *Bifidobacterium* spp., including *B. adolescentis* ATCC 15703 (accession no. AP009256)*, B. animalis* ATCC 25527 (accession no. CP002567), and *B. longum* NCC2705 (accession no. AE014295), were used as references for alignment in Projector 2 (10). Sanger sequencing for subsequent gap closing was performed at GATC-Biotech (Germany). Highly repetitive regions, such as clustered regularly interspaced short palindromic repeat regions (CRISPRs), were validated by primer walking and Sanger sequencing. Assembly was performed with Lasergene SeqMan Pro 8.0.2 (DNASTAR, Madison, WI) and CLC Genomics workbench 6.0.1 (CLC bio, Aarhus, Denmark). The genome was annotated using the NCBI Prokaryotic Genomes Automatic Annotation Pipeline (PGAAP, http://www.ncbi.nlm.nih.gov/genomes/static/Pipeline.html) and the Rapid Annotation using Subsystems Technology (RAST) platform (11) with RAST-based and Glimmer-3-based gene calling. Annotations obtained through these pipelines were compared and subsequently curated manually.

The genome of *B. thermophilum* RBL67 consists of a 2,291,643-bp circular molecule. The G+C content of the genome is 60.1%, which is within the 59.2 to 60.5% range of its relatives *B. adolescentis*, *B. animalis*, and *B. longum*. RBL67 harbors 47 tRNA genes and 12 rRNA genes, including 4 copies of the 16S rRNA gene. A total of 1,845 coding sequences (CDS) were predicted in the genome, of which 50 CDS have not been found so far in other *Bifidobacterium* species. However, 25 out of these 50 CDS were <100 amino acids. The complete genome of RBL67 will provide insight in the evolution of bifidobacteria and contributes to the further understanding of the biology of the genus and properties of this particular species.

### Nucleotide sequence accession number.

The genome sequence of *B. thermophilum* RBL67 was deposited at GenBank under the accession no. CP004346.
